# Effects of hypoglycemia on myocardial susceptibility to ischemia–reperfusion injury and preconditioning in hearts from rats with and without type 2 diabetes

**DOI:** 10.1186/s12933-017-0628-1

**Published:** 2017-11-09

**Authors:** Kim B. Pælestik, Nichlas R. Jespersen, Rebekka V. Jensen, Jacob Johnsen, Hans Erik Bøtker, Steen B. Kristiansen

**Affiliations:** Department of Cardiology, Aarhus University Hospital, Skejby Sygehus, Palle Juul-Jensens Blvd. 99, 8200 Aarhus N, Denmark

**Keywords:** Ischemia, Reperfusion, Infarct size, Glucose uptake, O-GlcNAc, Hypoglycemia, Diabetes

## Abstract

**Background:**

Hypoglycemia is associated with increased mortality rate in patients with diabetes. The underlying mechanisms may involve reduced myocardial tolerance to ischemia and reperfusion (IR) or reduced capacity for ischemic preconditioning (IPC). As IPC is associated with increased myocardial glucose uptake (MGU) during reperfusion, cardioprotection is linked to glucose metabolism possibly by O-linked β-N-acetylglucosamine (O-GlcNAc). We aimed to investigate the impact of hypoglycemia in hearts from animals with diabetes on myocardial IR tolerance, on the efficacy of IPC and whether modulations of MGU and O-GlcNAc levels are involved in the underlying mechanisms.

**Methods:**

In a Langendorff model using diabetic ZDF (fa/fa) and non-diabetic (fa/+) rats (n = 6–7 in each group) infarct size (IS) was evaluated after 40 min of global ischemia and 120 min reperfusion during hypoglycemia [(glucose) = 3 mmol/l] and normoglycemia [(glucose) = 11 mmol/l]. Myocardial glucose uptake and O-GlcNAc levels were evaluated during reperfusion. IPC was induced by 2 × 5 min of global ischemia prior to index ischemia.

**Results:**

IS increased in hearts from animals with (p < 0.01) and without (p < 0.01) diabetes during hypoglycemia compared to normoglycemia. IPC reduced IS during normoglycemia in both animals with (p < 0.01) and without (p < 0.01) diabetes. During hypoglycemia, however, IPC only reduced IS in hearts from animals with diabetes (p < 0.05). IPC increased MGU during reperfusion and O-GlcNAc levels in animals with diabetes during hypo- (MGU: p < 0.05, O-GlcNAc: p < 0.05) and normoglycemia (MGU: p < 0.01, O-GlcNAc: p < 0.05) and in animals without diabetes only during normoglycemia (MGU: p < 0.05, O-GlcNAc: p < 0.01).

**Conclusions:**

Hypoglycemia increases myocardial susceptibility to IR injury in hearts from animals with and without diabetes. In contrast to hearts from animals without diabetes, the hearts from animals with diabetes are amenable to cardioprotection during hypoglycemia. In parallel with IPC induced cardioprotection, MGU and O-GlcNAc levels increase suggesting that increased MGU and O-GlcNAc levels are involved in the mechanisms of IPC.

## Introduction

Type 2 diabetes mellitus (T2DM) increases morbidity and mortality after myocardial infarction (MI) [1, 2]. Albeit controversial, randomized controlled trials have suggested that an intensified glycemic control in patients with T2DM, with an increased incidence of hypoglycemia, is associated with increased mortality [3–5]. Hypoglycemia is a common adverse effect of treatment of T2DM with insulin and sulphonylureas [6]. Studies using continuous glucose monitoring systems have revealed a higher incidence of hypoglycemia than previously appreciated [7, 8]. The mechanistic link between hypoglycemia and increased cardiovascular mortality remains unclear. Reduced myocardial tolerance to ischemia and reperfusion (IR) or reduced capacity for activation of cardioprotection may be underlying mechanisms of increased mortality after MI in patients with T2DM. Cardioprotection can be activated by ischemic preconditioning (IPC), by which repetitive sublethal episodes of ischemia induce resistance towards myocardial IR injury [9]. Previous studies of IPC and myocardial IR susceptibility during hyperglycemic conditions have demonstrated attenuated efficacy of IPC compared to normoglycemia [10–12] while hyperglycemia per se, does not seem to influence on myocardial infarct size [13]. However, glucose fluctuations aggravate cardiac susceptibility to IR injury [14] suggesting that hypoglycemia may have impact on infarct size. An altered myocardial susceptibility to IR or capacity for cardioprotection during hypoglycemia may represent a mechanistic link between hypoglycemia and impaired outcome in patients with diabetes after MI.

A major mechanism involved in the influence of varying circulating glucose concentration on cardioprotection seems to involve the activity of hexosamine biosynthetic pathway (HBP), which is sensitive to changes in circulating glucose and glutamine concentrations [15], accordingly referred to as a “nutrient-sensing” pathway. Moreover, elevating O-linked β-N-acetylglucosamine (O-GlcNAc) glycocylation, the resultant of the HBP, promotes cell survival during cellular stress, whereas decreasing the levels of O-GlcNAc reduces cell survival [16]. O-GlcNAcylation seems associated with cardioprotection by IPC [17, 18]. We hypothesized that hypoglycemia modifies IR injury and capacity for cardioprotection by IPC differently in diabetic and non-diabetic hearts through simultaneous changes in myocardial glucose uptake and HBP activity during reperfusion.

The aims of the present study were to compare myocardial IR susceptibility, the cardioprotective efficacy of IPC and their associated changes in myocardial glucose uptake and O-GlcNAc levels in diabetic and non-diabetic during normo- and hypoglycemia.

## Materials and methods

### Animals

Experiments were conducted in 12 weeks old Zucker diabetic fatty (ZDF) rats [homozygote (fa/fa)] and their age-matched lean controls (fa/+) (Charles River Laboratories). Animals were housed under conditions maintained at 23 °C, 12 h light/dark cycles, 30% humidity, and allowed access to food (Purina 5008 diet) and water ad libitum. Housing was accomplished in dedicated facilities under inspection of animal technicians. The investigations conformed to Danish law for animal research (Act No. 1306 of 23/11/2007, Danish ministry of Justice) and the Guide for the Care and Use of Laboratory Animals published by the National Institute of Health.

### Protocols

**Fig. 1:**
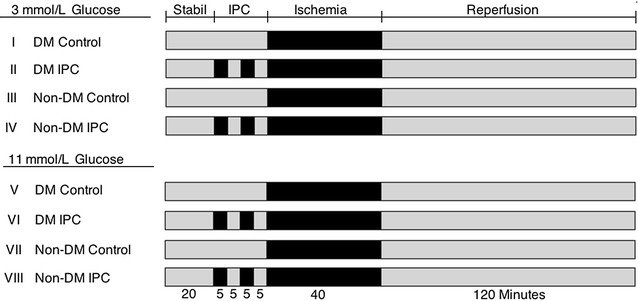
Experimental protocols. After stabilization (Stabil) ischemic preconditioning (IPC) was induced by two periods of 5 min of global ischemia followed by 5 min of reperfusion prior to ischemia in diabetic (DM) control, Non-DM control, DM IPC and Non-DM IPC hearts

Preceding the experiments, tail blood was sampled after 12 h fasting to validate development of T2DM by analyzing circulating blood glucose (OneTouch^®^ Ultra Blood Glucose, lifescan Inc., CA, USA) and insulin levels (AlphaLISA^®^ Insulin Kit, PerkinElmer, MA, USA). Animals were randomly allocated to eight experimental groups: I–IV 3 mmol/l glucose (n = 7 in each group) (I: DM control, II: DM IPC, III: Non-DM control, IV: Non-DM IPC) and V–VIII 11 mmol/l glucose (n = 6 in each group) (V: DM control, VI: DM IPC, VII: Non-DM control, VIII: Non-DM IPC), Fig. 1. Hypoglycemia at a glucose concentration of 3 mmol/l was used because baseline left ventricular developed pressure was preserved at this concentration. A glucose concentration of 11 mmol/l was used as normoglycemia because this is most commonly used concentration by researchers utilizing the Langendorff perfused heart model [19] and not considered hyperglycemic in the absence of free fatty acids and other substrates in the experimental model because normal postprandial glucose concentration in rat is up to 10.4 mmol/l [20]. All hearts were allowed to stabilize for 20 min. IPC hearts were subsequently preconditioned by 2 × 5 min of global ischemia. Each period of global ischemia was followed by 5 min of reperfusion. After 40 min all hearts were subjected to 40 min of global ischemia and 2 h of reperfusion.

### Isolated heart

An isolated perfused rat heart preparation was used as previously described [21]. Rats were anaesthetized by subcutaneous injection of Dormicum^®^ (0.5 mg of midazolam/kg of body weight; Matrix Pharmaceuticals, Herlev, Denmark) and Hypnorm^®^ (0.158 mg of fentanyl citrate/kg of body weight and 5 mg of fluanisone/kg of body weight, Vetapharma Ltd., Leeds, UK). A tracheotomy was performed and the rats were connected to a ventilation apparatus (Ugo Basile 7025 rodent ventilator, Comerio, Italy). The beating hearts were exposed through a thoracotomy and dissected from surrounding structures. The femoral vein was exposed by blunt dissection and a bolus of heparin 1.000 IU/kg (Leo Pharma, Copenhagen, Denmark) was injected. The hearts were cannulated in situ and retrograde perfusion was established with Krebs–Henseleit buffer (NaCl_2_ 118.5 mmol/l, KCl 4.7 mmol/l, NaHCO_3_ 25.0 mmol/l, glucosemonohydrate 3.0 or 11.0 mmol/l, MgSO_4_·7H_2_O 1.2 mmol/l, CaCl_2_ 2.4 mmol/l and KH_2_PO_4_ 1.2 mmol/l) at pH 7.4 and oxygenated with 5% CO_2_ and 95% O_2_. The perfusion buffer did not contain insulin as insulin per se exerts cardioprotection [22]. The hearts were rapidly excised, transferred to a Langendorff perfusion apparatus (IH-SR type 844/1; Hugo Sachs Electronik, Harvard Apparatus, March-Hugstetten, Germany) and perfused at constant pressure of 80 mmHg at 37 °C. A fluid-filled pressure balloon, connected to a pressure transducer, was placed in the left ventricle through the mitral valve. Coronary flow was measured using an in-line flow probe (Type 2.5SB, Transonic Systems Inc., Ithaca, NY, USA). Hemodynamic data were acquired and analyzed using dedicated software (Notocord Hem-v3.5, Croissy sur Seine, France).

### Myocardial infarct size

At the end of reperfusion hearts were frozen at − 80 °C for 15 min, sliced (≈ 1.5 mm), and stained with 1% 2.3.5-triphenyltetrazolium chloride, for 3 min at 37 °C and pH 7.4 as previously described [23]. After each slice was weighed and scanned (Epson Perfection V600, Epson, Nagano, Japan) the area of whole slice minus cavities, area at risk (AAR) and area of infarction (IS) were assessed by computer planimetry (UTHSCA ImageTool, San Antonio, TX, USA). IS/AAR was subsequently calculated and weighted with the weight of each slice weight. All measurements were done in a blinded fashion.

### Glucose uptake rate

Glucose uptake was assessed from rates of ^3^H_2_O production from D-[2-^3^H]-glucose (5 μCi/100 ml perfusate) [24]. Hearts were tracer perfused in the ion buffer from periods 10–40 and 80–110 min. Samples were collected as a baseline arterial sample (1 ml) and multiple effluent samples (1 ml), and immediately placed on ice and stored at − 80 °C. Glucose uptake rate was calculated as previously described [25] and presented as μmol × min^−1^ × g^−1^ dry weight.

### O-GlcNAc western blot

The heart apex was snap-frozen in liquid nitrogen immediately after end of reperfusion and weighed. Samples were thawed and homogenized in ice-cold extraction buffer added enzyme activity inhibitors (PIC2, PIC3, KF, B-glycerophosphate, TSA 1, Thiamet-G and PMSF). Protein concentration of the supernatant was determined using Pierce 660 nm Protein Assay (Thermo Scientific). Western blotting was performed by priming with antibodies: anti-O-GlcNAc antibody (CDT 110.6, Gift—CoreC4) and anti-actin antibody (Sigma). Densitometry was calculated relatively to densitometry of the corresponding actin blot bands. Results were presented as percentage of 3 mmol/l non-DM control (set at 100%).

### Statistical analysis

Analyses were blinded. Values are presented as mean ± SEM. Infarct-size, stabilization hemodynamics and glucose uptake, western blotting densitometry, blood-insulin, -glucose level, and rat weights were analyzed using one-way analysis of variance (ANOVA) with post-test when appropriate. Reperfusion hemodynamics was presented as left ventricular developing pressure (LVDP; LV systolic pressure—LV diastolic pressure) and rate pressure product (RPP; LVDP × rate) and compared using two-way ANOVA with repeated measurements. Calculations and artwork were performed using GraphPad Prism (GraphPad Software, LA Jolla, CA, USA). Two-sided p value < 0.05 was considered significant.

## Results

### Animal characteristics

Bodyweight was higher in animals with (366 ± 8 g) than without diabetes (303 ± 5 g) (p < 0.001). Overall, heart weight was higher in rats with than in rats without diabetes (p < 0.05) while heart weight/body weight ratio was smaller (p < 0.01). Most importantly hearts weights did not differ between compared groups (p = 0.08). Animals with diabetes had higher fasting blood glucose (9.9 ± 0.6 mmol/l vs. 4.6 ± 0.3 mmol/l, p < 0.001) and higher insulin concentrations (7.7 ± 1.6 μIU/ml vs. 0.7 ± 0.3 μIU/ml, p < 0.001) than animals without diabetes.

### Infarct size

**Fig. 2:**
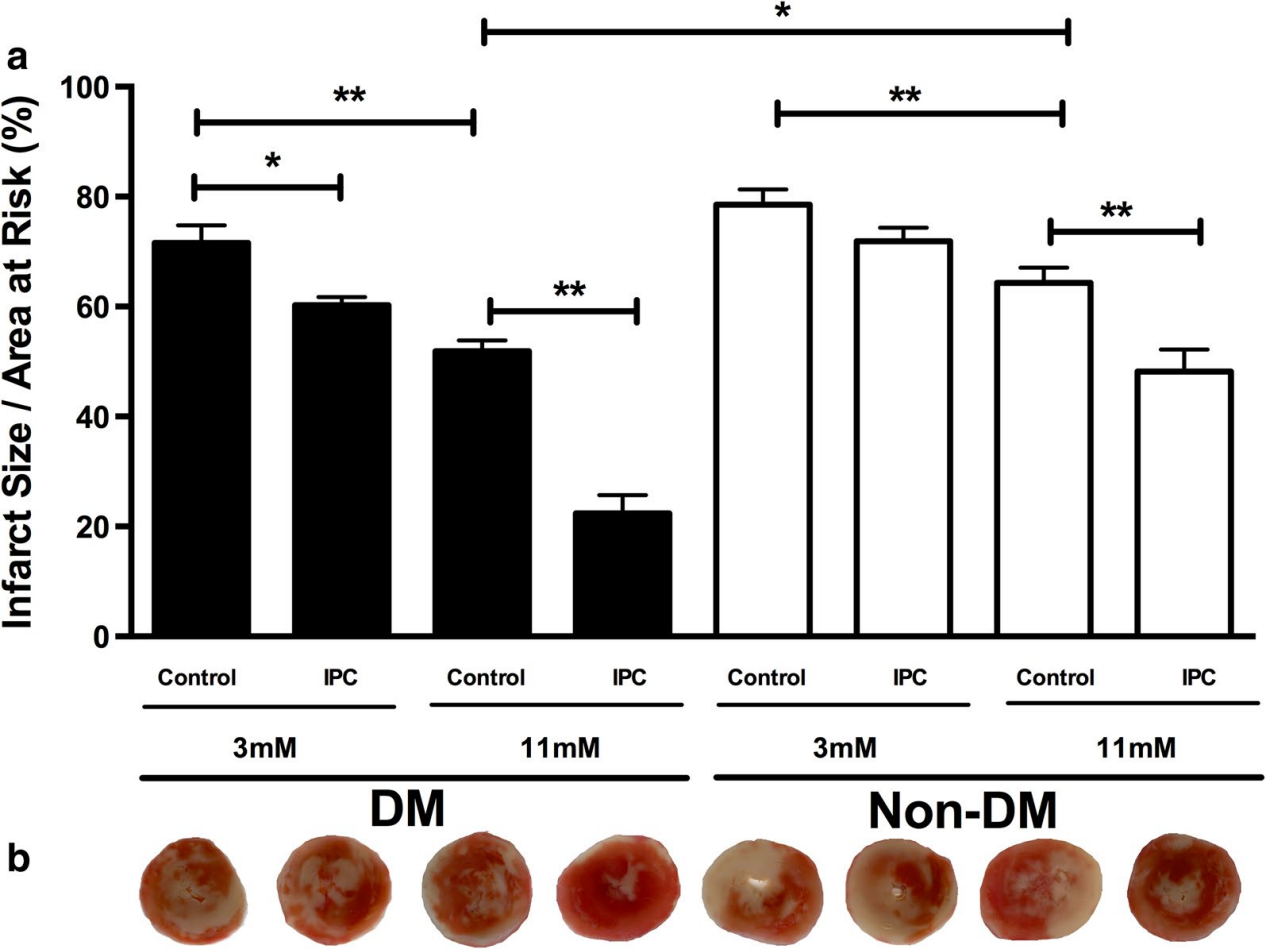
**a** Infarct-size/area at risk (%) at the end of 40 min of global ischemia and 120 min of reperfusion in diabetic (DM) control, Non-DM control, DM ischemic preconditioned (IPC) and Non-DM-IPC hearts. **b** Representative triphenyl tetrazolium chloride (TTC) stained sections for evaluation of infarct size in each group. Perfusion glucose level was 3 and 11 mmol/l. *p < 0.05; **p < 0.01. Mean ± SEM

Infarct size was smaller in animals with than without diabetes during normo- (p < 0.05) but not during hypoglycemia. Hypoglycemia increased myocardial infarct size in animals with (p < 0.01) and without diabetes (p < 0.01) compared with normoglycemia, Fig. 2. IPC reduced myocardial infarct size in animals with diabetes during normo- (p < 0.01) and hypoglycemia (p < 0.05) and in animals without diabetes during normo- (p < 0.01) but not during hypoglycemia. Area-at-risk did not differ between groups (p = 0.38).

### Hemodynamics

**Fig. 3:**
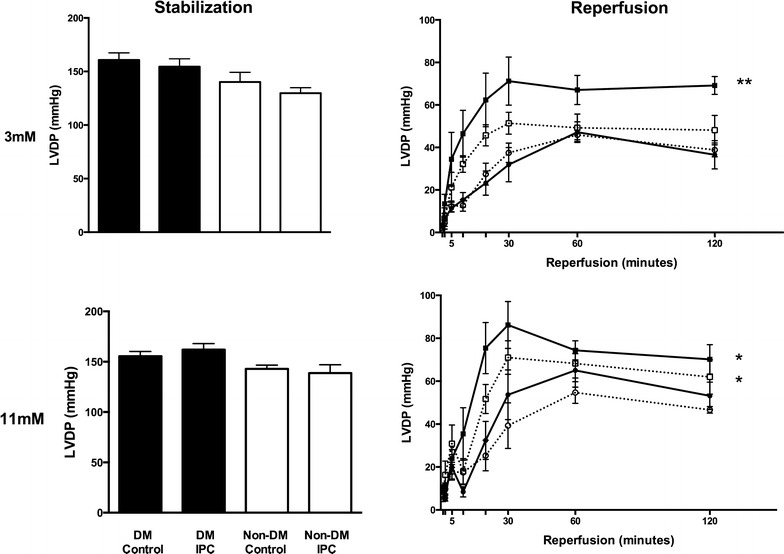
Left ventricular developed pressure (LVDP) in diabetic (DM) control ( ), DM ischemic preconditioned (IPC) ( ), Non-DM control ( ) and Non-DM IPC ( ) hearts during stabilization and reperfusion. Perfusion glucose level was 3 and 11 mmol/l. *p < 0.05; **p < 0.01 compared to control. Mean ± SEM

Whereas LVDP did not differ between animals with and without diabetes during stabilization and reperfusion at both glucose levels, RPP was decreased in hearts from animals with diabetes compared to without diabetes during stabilization at hypoglycemia (p < 0.01), Fig. 3, Table 1. Hypoglycemia reduced RPP in diabetic controls during reperfusion (p < 0.05 vs. normoglycemia) (Table 1). IPC increased LVDP and RPP during reperfusion at normoglycemia in both hearts from animals with (LVDP: p < 0.05, RPP: p < 0.01) and without diabetes (LVDP: p < 0.05, RPP: p < 0.05) animals. IPC increased LVDP and RPP during hypoglycemia only in animals with diabetes (LVDP: p < 0.01, RPP: p < 0.05). Coronary flow was increased by IPC during reperfusion in animals with diabetes during hypoglycemia (p < 0.05) and in animals without diabetes during normoglycemia (p < 0.01) compared to controls, Table 1.

**Table 1:** Rate pressure product and coronary flow before and after ischemia

	Stabilization	Ischemia	Reperfusion							
	20 min		1 min	2 min	5 min	10 min	20 min	30 min	60 min	120 min
RPP (mmHg × min × 100)										
3 mM DM control	164 ± 18^d^	–	7 ± 2	12 ± 4	20 ± 4	26 ± 7	33 ± 7	46 ± 9	82 ± 20	60 ± 13^c^
3 mM DM IPC	178 ± 17	–	7 ± 2	19 ± 6	45 ± 15	60 ± 18	77 ± 15	90 ± 22	123 ± 19	127 ± 17^a^
3 mM Non-DM control	302 ± 24	–	4 ± 1	9 ± 4	30 ± 7	31 ± 7	73 ± 17	86 ± 13	106 ± 13	85 ± 13
3 mM Non-DM IPC	267 ± 10	–	4 ± 1	14 ± 10	44 ± 17	57 ± 9	72 ± 5	90 ± 7	89 ± 8	89 ± 12
11 mM DM control	210 ± 34	–	27 ± 4	21 ± 7	45 ± 12	20 ± 6	39 ± 9	91 ± 22	109 ± 12	87 ± 14
11 mM DM IPC	249 ± 42	–	19 ± 5	14 ± 2	59 ± 9	75 ± 18	134 ± 21	142 ± 23	200 ± 19	145 ± 14^b^
11 mM Non-DM control	289 ± 32	–	18 ± 2	18 ± 6	36 ± 6	31 ± 9	42 ± 9	72 ± 20	93 ± 16	86 ± 10
11 mM Non-DM IPC	270 ± 31	–	12 ± 4	33 ± 15	94 ± 30	41 ± 14	127 ± 24	152 ± 28	173 ± 27	176 ± 27^a^
Flow (ml/min)										
3 mM DM Control	14 ± 1	–	16 ± 1	15 ± 1	14 ± 1	16 ± 1	17 ± 1	16 ± 1	14 ± 1	13 ± 1
3 mM DM IPC	15 ± 1	–	21 ± 1	18 ± 2	19 ± 1	20 ± 1	20 ± 1	19 ± 2	18 ± 2	15 ± 1^a^
3 mM Non-DM Control	18 ± 1	–	14 ± 1	13 ± 1	14 ± 1	16 ± 1	17 ± 1	17 ± 1	15 ± 1	12 ± 2
3 mM Non-DM IPC	15 ± 1	–	15 ± 2	14 ± 1	15 ± 2	16 ± 2	15 ± 1	15 ± 1	13 ± 1	11 ± 1
11 mM DM Control	16 ± 1	–	18 ± 2	16 ± 2	17 ± 2	17 ± 2	17 ± 1	16 ± 2	14 ± 2	12 ± 1
11 mM DM IPC	18 ± 1	–	22 ± 3	19 ± 2	20 ± 2	20 ± 3	20 ± 2	20 ± 3	19 ± 3	15 ± 3
11 mM Non-DM Control	18 ± 1	–	15 ± 2	13 ± 1	14 ± 1	15 ± 1	14 ± 1	13 ± 1	13 ± 1	10 ± 1
11 mM Non-DM IPC	20 ± 2	–	23 ± 1	20 ± 1	22 ± 1	23 ± 1	23 ± 1	21 ± 1	17 ± 2	15 ± 1^b^

Data are mean ± SEM

*Non-DM* non diabetic rats, *DM* diabetic rats, *RPP* rate pressure product

^a^p < 0.05 compared to corresponding control

^b^p < 0.01 compared to corresponding control

^c^p < 0.05 compared to corresponding normoglycemic control

^d^p < 0.01 compared to Non DM control

### Myocardial glucose uptake

**Fig. 4:**
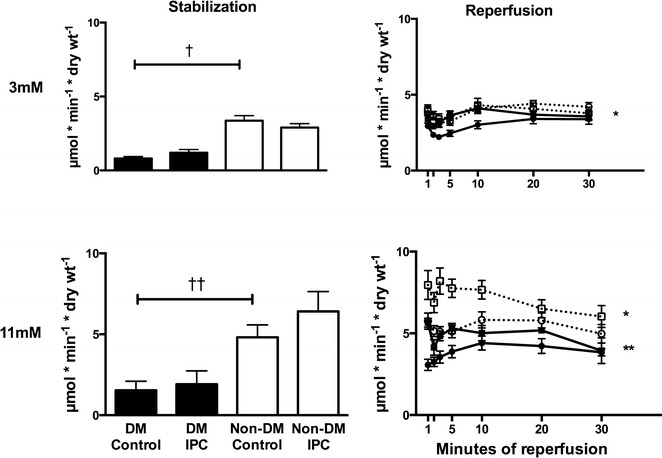
Tracer-estimated exogenous glucose uptake in diabetic (DM) control ( ), DM ischemic preconditioned (IPC) ( ), Non-DM control ( ) and Non-DM IPC ( ) hearts during stabilization and reperfusion. Perfusion glucose level was 3 and 11 mmol/l. ^†^p < 0.05; ^††^p < 0.01; *p < 0.05 compared to control; **p < 0.01 compared to control. Mean ± SEM

Myocardial glucose uptake was lower in animals with diabetes than without diabetes during hypo- and normoglycemia at both stabilization and reperfusion, Fig. 4. Hypoglycemia reduced myocardial glucose uptake in animals with (p < 0.01 vs. normoglycemia) and without diabetes (p < 0.01 vs. normoglycemia) during reperfusion. IPC increased myocardial glucose uptake in normoglycemic animals with (p < 0.01) and without diabetes (p < 0.05) during reperfusion while a similar increase was only seen in animals with diabetes during hypoglycemia (p < 0.05).

### Myocardial O-GlcNAc concentrations

**Fig. 5:**
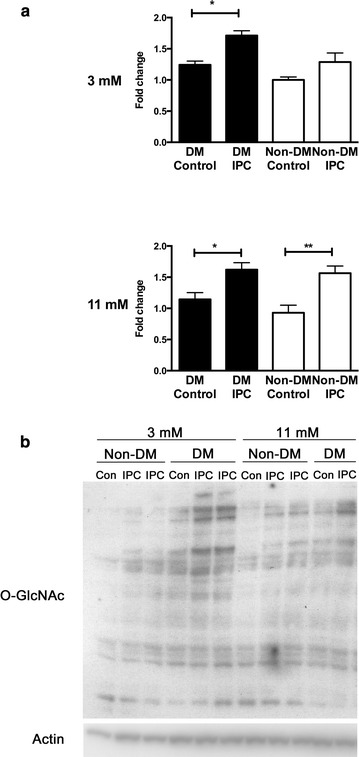
**a** O-GlcNAc (CTD110.6 antibody) levels in diabetic (DM) control, Non-DM control, DM ischemic preconditioned (IPC) and Non-DM IPC hearts expressed as fold change compared to 3 mmol/l Non-DM control and correlated against actin. **b** Representative O-GlcNAc and actin immunoblots. Note the higher intensity of the O-GlcNAc bands in the preconditioned compared with corresponding control hearts. *p < 0.05, **p < 0.01. Mean ± SEM

Myocardial levels of *O*-GlcNAc were similar in animals with and without diabetes during hypo- and normoglycemia, Fig. 5. Hypoglycemia induced no changes compared to normoglycemia in animals with or without diabetes (Fig. 5). IPC increased *O*-GlcNAc levels in animals with diabetes during both normoglycemia (p < 0.05) and hypoglycemia (p < 0.05) but only during normoglycemia (p < 0.01) in animals without diabetes.

## Discussion

The present study demonstrates that myocardial susceptibility to IR is augmented during hypoglycemia in both rats with and rats without diabetes. However, the cardioprotective effect of IPC is preserved during hypoglycemia in rats with diabetes hearts contrary to rats without diabetes. The underlying mechanisms of IPC induced cardioprotection are associated with increased myocardial glucose uptake and O-GlcNAc levels in animals with diabetes as well as without diabetes. Increased myocardial susceptibility to IR during hypoglycemia may be a mechanistic link between hypoglycemia and impaired outcome after MI in patients with diabetes.

Myocardial susceptibility to IR in diabetic hearts remains controversial because studies in animal models of type 1 and 2 diabetes have yielded conflicting results [21, 26–32]. The discrepancy may be explained by different species and models and also by the age of animals and duration of diabetes [25]. In the present study, we confirm our previous findings of reduced susceptibility to IR during normoglycemia in a T2DM animal model with recent onset of diabetes [21, 25]. Our findings, that infarct size was increased in both hearts from animals with and without diabetes during hypoglycemia and that no difference in infarct size was observed between animals with and without diabetes, are supported by previous findings of increased IR susceptibility in animals without diabetes during hypoglycemia in the brain [33] and heart [34]. We now extended these findings to a clinically more relevant model of hypoglycemia in animals with diabetes. The absent difference in infarct size between animals with and without diabetes during hypoglycemia indicates that the endogenous cardioprotection observed in hearts from animals with diabetes during normoglycemia at onset of diabetes [25, 35] seems to be lost during hypoglycemia. Together, our findings emphasize the importance of glucose concentration during IR, when evaluating myocardial susceptibility to IR in hearts from animals with or without diabetes. Myocardial susceptibility to IR during hypoglycemia is of particular importance in diabetic hearts because patients with diabetes frequently suffer episodes of hypoglycemia due to treatment with glucose lowering drugs.

In contrast to our previous findings [21], IPC reduced myocardial infarct size and improved post ischemic left ventricular function in both animals with and without diabetes during normoglycemia. The diabetic heart may still be amenable to protection by IPC with an intensified stimulus that overcomes the increased threshold for the necessary activation of pro-survival kinases [30]. Consequently, we used a more aggressive stimulus by two cycles of 5-min ischemia and 5-min reperfusion in contrast to four cycles of 2-min ischemia followed by 3-min reperfusion as used previously [21]. IPC retained cardioprotection during hypoglycemia in diabetic hearts by reducing infarct size, albeit to a lesser extent than during normoglycemia. In contrast, the cardioprotective effect of IPC in non-diabetic animals during hypoglycemia was abolished.

Because osmolarity changes with variations in circulating glucose concentrations, changes in osmolarity may influence infarct size. A change in plasma glucose from 11 to 3 mmol/l would lead to a 2.5% reduction in osmolarity of the perfusion buffer. The influence of osmolarity on infarct size has varied in previous studies. Kersten et al. [36] demonstrated that increases in serum osmolarity obtained by administration of raffinose did not influence infarct size or interfere with the ability of IPC to protect against infarction. In contrast, Zalesak et al. showed that a hyperosmotic environment blunted the efficiency of IPC against IR injury and improved ischemic tolerance in non-preconditioned isolated rat hearts suggesting that increased osmolarity, similar to that in the hyperglycemic conditions, may play a pivotal role in a failure of IPC to induce cardioprotection in the diabetic myocardium [37]. Since glycemic levels and osmolarity were altered similarly by hypoglycemia and infarct size was equally increased in animals with and without diabetes, we cannot establish whether the increment was caused by hypoglycemia or a reduction in osmolarity. However, an increment in infarct size due to reduced osmolarity would be discrepant to previous findings. The different responses to IPC in animals with and without diabetes during hypoglycemia cannot be related to the minor reductions in osmolarity as the reductions were identical in the two groups.

Consistent with previous findings during non-ischemic conditions [25, 38], we report reduced myocardial glucose uptake during reperfusion and stabilization in hearts from animals with diabetes. Stimulation of glycolysis and glucose oxidation during reperfusion improves post-ischemic left ventricular functional recovery [39–41]. Cardioprotection by IPC is associated with increased myocardial glucose uptake during reperfusion in non-diabetic hearts [41, 42] as confirmed in hearts from animals without diabetes during normoglycemia in the present study. However, the absent infarct sparring effect of IPC in hearts from animals without diabetes during hypoglycemia was associated with an absent modulation of myocardial glucose uptake during reperfusion by IPC in animals without diabetes. In contrast, IPC increased myocardial glucose uptake during reperfusion in hearts from animals with diabetes at normo- as well as hypoglycemia. These findings parallel cardioprotection afforded by IPC in hearts from animals with diabetes during normo- and hypoglycemia. Together, our findings support the notion that an underlying mechanism of the effects behind IPC involves myocardial glucose uptake as only IPC generated increments in myocardial glucose uptake translated into cardioprotection. This notion is further supported by findings of increased myocardial glucose uptake associated with improved functional recovery after ischemia in animals with diabetes treated with rosiglitazone [43].

O-linked β-N-acetylglucosamine glycocylation is a recently detected posttranslational modification of nuclear, cytoplasmic and mitochondrial proteins [44]. *O*-GlcNAc acts as an intracellular stress sensor, linking glucose metabolism to cellular function at a molecular level [15, 45]. O-GlcNAcylation is dependent upon substrate synthesis in the HBP [46]. Flux through the HBP parallels glucose availability [15, 46] and increased glucose uptake is linked to HBP activation and downstream formation of O-GlcNAc [47]. On the molecular level, O-GlcNAcylation is implicated as a major mechanism of glucose toxicity and insulin resistance in diabetes [48, 49]. In the present study investigating myocardial O-GlcNAc levels during reperfusion, we did not observe differences between animals with and without diabetes, which may be related to the short duration of diabetes in the relatively young animals used in the present study. Augmentation of cardiac O-GlcNAc levels by glucosamine or salidroside administration affords cardioprotection [50, 51]. In addition, O-GlcNAc has been suggested to be involved in the underlying mechanism of IPC [17, 18]. The mechanisms by which acute elevation of O-GlcNAc levels induce cardioprotection seem to remain intact in the hearts from animals with diabetes even during hypoglycemia as IPC induced reduction in infarct size was associated with increased myocardial O-GlcNAc levels in animals with diabetes during both normo- and hypoglycemia. However, in hearts from animals without diabetes, IPC did not reduce infarct size or increase myocardial glucose uptake and did not influence O-GlcNAc levels during hypoglycemia. Accordingly, increased myocardial O-GlcNAc levels and glucose uptake may represent at mechanistic link to cardioprotection afforded by IPC.

The clinical implication of our findings is that myocardial infarct size in patients with T2DM may be larger during hypoglycemia than during normoglycemia similarly to the animals investigated in the present study. This finding may explain the observed increased mortality in clinical trials investigating an intensified glycemic control in patients with T2DM [3–5], as myocardial infarct size is a prognostic factor after MI [52]. However, infarct size alone does not seem to be the only determining mechanism of the overall higher mortality in patients with T2DM suffering an acute MI compared with patients without diabetes as infarct size at identical glucose concentrations was smaller or equally sized in animals with and without T2DM in the present study.

Limitations of the present study primarily relate to the use of an isolated perfused heart model but we used this to investigate the impact of hypoglycemia on myocardial susceptibility to IR and not the combined effects of hypoglycemia and a blood glucose lowering compound. However, in vivo induction of hypoglycemia by insulin, sulphonylureas or other agents may influence findings, as these agents per se impacts the effects of IR and IPC [53, 54]. Moreover, an uncontrolled systemic response to hypoglycemia, including activation of neurogenic and humoral components, may constitute important limitations. Importantly, such effects can be excluded in our model. We choose specifically to investigate the effects of hypoglycemia on infarct size in a glucose dependent model in the absence of free fatty acids to specifically investigate the impact on MGU without interference from other substrates. However, the presence of free fatty acids as well as other substrates may potentially have influenced our findings. We used a glucose concentration of 11 mmol/l as this is most commonly used concentration in the Langendorff perfused heart model [19]. Because normal postprandial glucose concentration in the rat is up to 10.4 mmol/l [20] a glucose concentration of 11 mmol/l is not considered hyperglycemic in the absence of free fatty acids and other substrates in the experimental model. We used relatively young animals with a short duration of diabetes. This may potentially limit the generalizability to patients with long lasting T2DM.

In conclusion, hypoglycemia increases myocardial infarct size in hearts from animals with and without diabetes. In contrast to hearts from animals without diabetes, hearts from animals with diabetes are amenable to cardioprotection during hypoglycemia. Increased myocardial glucose uptake and O-GlcNAc levels seem to be involved in the cardioprotective mechanisms of IPC irrespective of circulating glucose concentrations.

## Abbreviations

AAR: area at risk
ANOVA: analysis of variance
DM: diabetes mellitus
HBP: hexosamine biosynthetic pathway
IPC: ischemic preconditioning
IR: ischemia reperfusion
IS: area of infarction
LVDP: left ventricular developing pressure
MGU: myocardial glucose uptake
MI: myocardial infarction
O-GlcNAc: O-linked β-N-acetylglucosamine
RPP: rate pressure product
T2DM: type 2 diabetes mellitus
ZDF: Zucker diabetic fatty
